# Towards an Ultra Sensitive Hybrid Mass Sensor Based on Mode Localization without Resonance Tracking

**DOI:** 10.3390/s20185295

**Published:** 2020-09-16

**Authors:** Claude Humbert, Vincent Walter, Najib Kacem, Thérèse Leblois

**Affiliations:** FEMTO-ST Institute, University Bourgogne Franche-Comté, CNRS/UFC/ENSMM/UTBM, 25000 Besançon, France; vincent.walter@femto-st.fr (V.W.); najib.kacem@femto-st.fr (N.K.); therese.leblois@femto-st.fr (T.L.)

**Keywords:** mode localization, mass sensing, QCM, FPGA, hybrid system, open loop, fixed frequency

## Abstract

We present a mode localized mass sensor prototype based on a hybrid system excited at a fixed frequency slightly below the resonances. Indeed, we show, both theoretically and experimentally, that this condition yields higher sensitivities and similar sensitivity ranges than that of resonance peak tracking while being less time consuming than a classical open-loop configuration due to the absence of frequency sweep. The system is made of a quartz resonator and a hardware that includes a resonator and the coupling. The digital aspect allows maximum sensitivity to be achieved with a fine tuning of the different parameters and the implementation of a coupling, regardless of the physical resonator geometry. This allows the generation of mode localization on shear waves resonant structures such as the quartz cristal microbalance widely used in biosensing. This solution has been successfully implemented using resin micro balls depositions. The sensitivities reach almost their maximum theoretical values which means this fixed frequency method has the potential to reach lower limit of detection than the open loop frequency tracking method.

## 1. Introduction

The last two decades saw the development of sensors that were based on arrays of weakly coupled resonators. Using two or more weakly coupled resonators allows for taking advantage of the mode localization (ML) phenomenon, which is a manifestation of the well-known Anderson localization [1] applied to structural dynamics, often described, as follows: in a weakly coupled symmetrical system, the introduction of a slight perturbation breaking the symmetry of the structure will cause a drastic confinement of the vibrational energy.

The output parameter of mode localized sensors is the vibration amplitude shift, whether for evaluating a change in eigenvectors or amplitude ratios at resonance. This is a major difference from mechanical resonant sensors that measure a change in resonant frequency (RtF). While the resolution of such sensors is rather good, the normalized sensitivity (NS), defined as the relative output over input shifts, is limited to the constant value of $\frac{1}{2}$ [2]. On the other hand, the theoretical NS of mode-localized sensors can be two to three orders of magnitude higher than this value. The lower the coupling, the higher the NS. However, there is a low limit for weak coupling imposed by the mode aliasing that appears when the frequency difference between two vibration modes is too small with respect to the bandwidth of the modes, so that the two modes merge [3]. Therefore, it seems appropriate to work with high quality factor resonators to achieve the highest possible NS.

Most of the papers dealing with mode localized sensors concern MEMS sensors. These sensors have been developed for various applications, ranging from mass sensors [4,5,6] to force [7] or acceleration [8,9] sensors, electrometers [10,11,12], and magnetometers [13,14].

The main disadvantage of MEMS sensors using ML is manufacturing defects. These defects make it difficult to produce perfectly identical resonators, which is a necessary condition for obtaining a balanced system before perturbation. One strategy to counteract this is to use electrostatic actuation in order to use electrostatic softening to rebalance the system after manufacturing [15]. Another disadvantage of MEMS sensors with ML is the lack of adjustment of the coupling, which does not necessarily allow for reaching the optimal value leading to the highest possible normalized sensitivity. In the case of mechanical coupling, the coupling value is directly dictated by the geometry of the coupling structure. Therefore, some sensors use electrostatic coupling, which allows for adjustment by varying the voltage [5,6,16,17], but prevents the sensor from being used in a liquid medium. However, such coupled structures cannot be designed using high Q-factor shear waves resonators, such as the quartz cristal microbalance (QCM), because of their geometry and wave form.

Here, we present an alternative solution based on a hybrid system, where a QCM is connected to a field programmable gate array (FPGA) that emulates the presence of a second virtual and tunable coupled resonator, in order to overcome these limitations. In such an architecture, maximum sensitivity can be achieved and geometry constraints due to the coupling are suppressed. Tunable ML has already been demonstrated on electrical resonators [18] and a device following the same principle has also been recently presented, where a cantilever is virtually coupled with an electrical resonator made of passive and active components to achieve sensitive mass sensing by means of ML [19]. Finally, in previous publications were shown the principle of virtualization [20,21], where only digital perturbations were applied.

This work first exposes the theoretical results in Section 2 (analytical developments and simulations) on NS in a two degrees of freedom (DoF) coupled resonators subjected to a mass perturbation. It also introduces a new open loop sensing method based on the amplitude shift at a fixed excitation frequency and discuss its advantages and drawback in regards with the classic method that consists in the vibrations amplitude measurements at the resonances. The measure of a variation in vibration amplitude due to a RtF shift is already exploited in atomic force microscopy for instance [22]. The concept of hybrid system along with its design are detailed in Section 3. It includes a description of the digital filter, the analog resonant filter based on a QCM and the complete hybrid system. Section 4 gives experimental results that confirm the theoretical ones that are presented in Section 2 by the means of particle depositions on the QCM of our system. It also gives a tuning protocol and a description of the experiments. These results are finally discussed in Section 5, where many perspectives are also exposed.

## 2. Theoretical Developments

We first demonstrate that exciting a pair of coupled resonators at a fixed excitation frequency (lower frequency of the resonance bandwidth) yields higher amplitude sensitivities to mass perturbations than the classic method, which consists in tracking the resonances. To do so, we provide analytical developments and simulation results on the maximum reachable NS and the sensitive range according to the Q-factor of the resonators in a two DoF weakly coupled resonators system. The sensitive range is here defined as the normalized perturbation at which the NS drops by half. Both resonators are modeled by the classic linear mass-spring (undamped resonator) or mass-spring-damper (damped resonator) in the analytical developments in order to provide general knowledge on ML. The proof of Properties 1 and 2 are given in Appendix A.

**Property** **1.** *Expression of ns_1_, the maximum normalized sensitivity (NS) in amplitude following the resonance in a 2 DoF undamped resonators system taking mode aliasing into account.*

(1) $$ns_{1}≃0.25\times Q$$

*where Q is the Q-factor of the damped resonator.*

**Property** **2.** *Expression of ns_2_, the NS in amplitude at fixed excitation frequency $f_{1}=f_{r}\;\cdot \;\left(1-\frac{1}{2Q}\right)$ for a single damped resonator of resonant frequency $f_{r}$.*

(2) $$ns_{2}≃0.35\times Q$$

*where Q is the Q-factor of the damped resonator.*

Properties 1 and 2 show that exciting a single resonator at the frequency $f_{1}$ (the RtF minus half the bandwidth) enables reaching maximum sensitivity to mass perturbation, most likely in a limited sensitive range. Because $ns_{2}>ns_{1}$, there is apparently no sensitivity gain when using a two DoF weakly coupled resonators system. However, it could be considered to exploit both phenomena at the same time: exciting a coupled structure at $f_{1}$ should indeed enable to observe a signal variation due to both ML and the RtF downshift.

**Figure 1:**
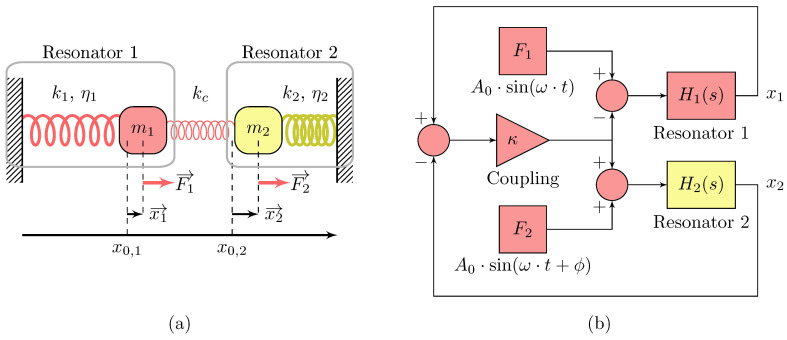
Two coupled resonators having linear stiffness $k_{i}$ and damping $\eta_{i}$, where $\kappa =\frac{k_{c}}{k_{1}}$. (**a**): Mass-spring like coupled resonators (**b**): Equivalent block diagram of (**a**).

We now demonstrate the two results from Properties 1 and 2 by the mean of Matlab^®^2016.b simulations on coupled and uncoupled damped resonators; the models are given in Figure 1 and Equation (3).

(3) $$H\left(s\right)=\frac{1}{\left(1+\epsilon \right)\;\cdot \;s^{2}+\frac{1}{Q}\;\cdot \;s+1}$$

The perturbation $\epsilon =\frac{\delta m}{m}$ is only applied on resonator 2. These simulations enable comparing the sensitive range of both methods. The NS are computed for a range of coupling $\kappa =\frac{k_{c}}{k_{1}}$ and mass perturbation ϵ, for each mode *i* and resonator *j*. Each mode of each resonator is tracked in order to calculate this NS, following its definition

(4) $$ns\left(i,j,Q,\kappa ,\epsilon \right)=\frac{1}{x_{r,\epsilon =0,\kappa =0}\left(Q\right)}\;\cdot \;\frac{\partial x_{r}}{\partial \epsilon }\left(i,j,Q,\kappa ,\epsilon \right)$$

where $x_{r,\epsilon =0,\kappa =0}$ and $x_{r}$ are the resonance amplitudes before mass perturbation and without coupling, and after mass perturbation, respectively.

**Figure 2:**
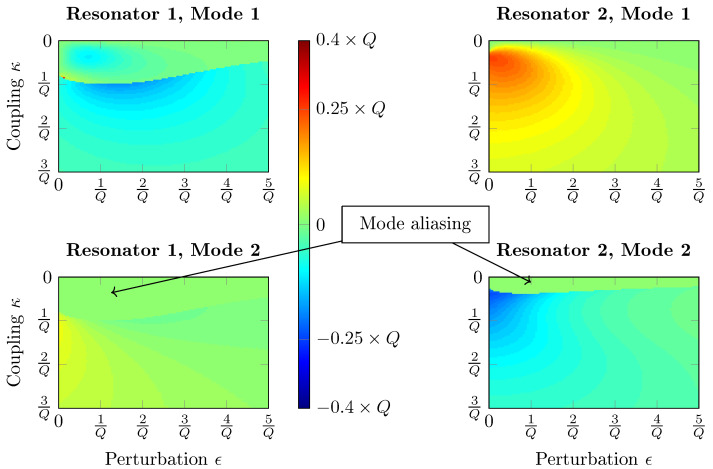
NS graphics of a 2 DoF damped resonators system with a mass perturbation on resonator 2. Output metrics: Resonance amplitude shift. The reference amplitude is the resonant amplitude of a single resonator.

**Figure 3:**
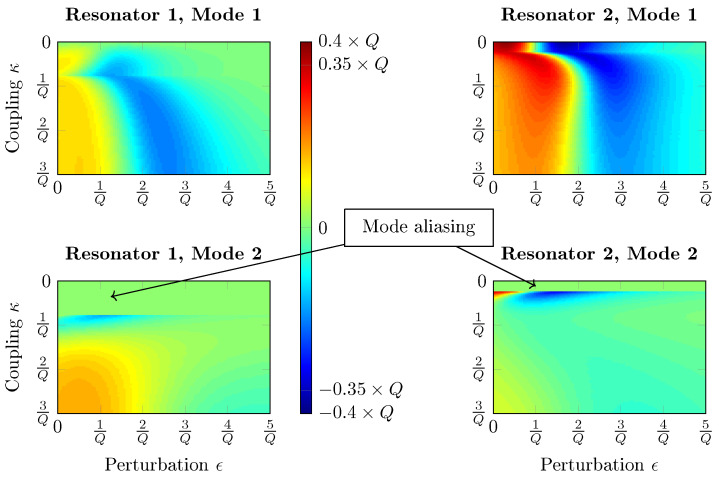
NS graphics of a two DoF damped resonators system with a mass perturbation on resonator 2. Output metrics: Amplitude shift at $f_{1}=f_{r}\;\cdot \;\left(1-\frac{1}{2Q}\right)$ for both resonances. The reference amplitude is the resonant amplitude of a single resonator.

Each of the graphs from Figure 2 and Figure 3 should be read line by line, from left to right, which is for a fixed coupling value κ and increasing mass perturbation ϵ. Hot and cold colors represent a signal increase and decrease, respectively. Figure 2 depicts the NS of a 2 DoF damped resonators system where both resonators are excited, with a phase of 90 degrees on the second resonator so that both modes appear in the frequency response. The first observation is that these sensitivities, perturbations and couplings are linked by the Q-factor. Indeed, the same graphs are obtained for different scales, as long as Q≫1. The second observation is the presence of mode aliasing that indeed prevents the sensitivity from spiking. This phenomenon does not appear exactly at the same coupling value because an anti-resonance between both modes appears on resonator 2 due to the excitation phase of 90 degrees. The observed maximum sensitivity is $|ns_{max}|=\frac{Q}{4}$, which is consistent with Property 1. Finally, it can be observed that the NS decreases rapidly when either κ or ϵ increase, a known property of ML.

The NS of amplitude shift at the fixed frequency $f_{1}$ (Figure 3) has also been computed with Matlab^®^for the damped resonators system. These simulations show that the NS value for κ=0 and ϵ=0 is −0.35×Q, as predicted by Property 2. Moreover, one can observe the first mode of the second resonator without coupling (κ=0, case of a single resonator subjected to a mass perturbation). The amplitude first increases until the resonance reaches $f_{1}$ at $\epsilon =\frac{1}{Q}$. This perturbation value doubles when the system is not subjected to mode aliasing (around $\kappa =\frac{0.5}{Q}$), since the RtF decreases with a NS twice lower because of the coupling, $\frac{1}{4}$ instead of $\frac{1}{2}$ (these values can be calculated from Property A1 in Appendix A). As a consequence, the amplitude gain due to the RtF downshift decreases by half as well when there is no mode aliasing, which is balanced by the effect of ML.

In conclusion, it can be stated that the normalized sensitivity in amplitude variation, measured at a fixed frequency for a single resonator, is slightly higher than that of a weakly coupled system with two DoF. This result calls into question the relevance of the use of mode localization from the point of view of sensitivity. The sensitive range of coupled systems is around $\frac{2}{Q}$ both if the resonances are tracked or at the fixed excitation frequency $f_{1}$, which makes the fixed frequency method worth investigating experimentally. Therefore, both methods are experimentally tested here with our high Q-factor hybrid system.

## 3. Materials and Methods

### 3.1. Concept of the Hybrid System

The idea behind a hybrid weakly coupled resonators system lays on the replacement of mechanical and non-tunable components by non mechanical but tunable elements in a classic MEMS array. Implementing ML on piezoelectric resonators provides an interesting approach, since the electro mechanical transduction is naturally done with such materials in both ways.

When considering a transfer function approach, both of the resonators and the coupling contribution can be separated. Therefore, if a piezoelectric resonator, such as a QCM, could be integrated in an electrical circuit with two terminals, an input and an output, the coupling contribution could be simply replaced by a signal processing in closed loop such as depicted in Figure 1, where the yellow part represents the mechanical resonator on which the mass perturbation is introduced and the red part are functions implemented in a hardware. In this way, the coupling value could indeed be easily tuned and also implemented with any kind of piezoelectric resonator, independently from its geometry.

### 3.2. Mathematical Tools

Digital filters do not have the limitations of electrical filters: any polynomial transfer function can be implemented and all of their coefficient can be chosen and finely tuned with no drift due to ambient conditions. In addition, a hardware can host a routine for signal recording, data processing, graphical user interface (GUI), and so on. However, the use of a hardware requires an appropriate mathematical tool to describe the sampled dynamic behavior of the system: the Z-transform of complex variable *z* can be seen as the discrete equivalent of the Laplace transform of complex variable *p*, which is broadly used in the continuous system analysis. This equivalence is done through the formula:

(5) $$z=e^{\frac{p}{f_{s}}}$$

where $f_{s}$ is the sampling frequency. Therefore, Equation (5) introduces non-polynomial transfer function from the Laplace transforms of a dynamic system. Knowing that in sinusoidal excitation of angular frequency ω, p=j·ω and $\omega \ll 2\pi \;\cdot \;f_{s}$, there is $\left|\frac{p}{f_{s}}\right|\ll 1$. This, Equation (5) can be approximated, which has the drawback to distort frequencies, a phenomenon called warping [23]. Therefore, a pre-warp bilinear transform allows to compensate this shift at a given angular frequency $\omega_{0}$. The filter response then follows that predicted by the continuous model around this particular frequency. The expression of the normalized Laplace variable $s=j\;\cdot \;\frac{\omega }{\omega_{0}}$ for the pre-warp bilinear transform is given by

(6) $$\begin{array}{c} s=\frac{1}{\tan\left(\frac{\omega_{0}}{2f_{s}}\right)}\;\cdot \;\frac{z-1}{z+1} \end{array}$$

The use of the system Laplace transform and Equation (6) yield the Z-transform of the system transfer function Z(H):

(7) $$\begin{array}{cc} Z(H) & =\frac{\sum_{k=0}^{n}\alpha_{k}\;\cdot \;z^{-k}}{\sum_{k=0}^{n}\beta_{k}\;\cdot \;z^{-k}} \end{array}$$

where $\beta_{0}\neq 0$.

Denoting $S_{i}$ the output and $E_{i}$ the input of the filter for a given time sample *i*, the previous equation can also be written as a linear combination of the input, previous inputs and outputs, as follows:

(8) $$\begin{array}{cc} S_{i} & =\sum_{k=0}^{n}a_{k}\;\cdot \;E_{i-k}-\sum_{k=1}^{n}b_{k}\;\cdot \;S_{i-k} \end{array}$$

When $b_{k}=0$, ∀k∈{1…n}, the output only depends on the input. Such filters are called finite impulse response filters (FIR). In contrast, if ∃k∈{1…n}, $b_{k}\neq 0$, the filter is called an infinite impulse response filter (IIR). Dynamic systems are usually IIRs, which demands careful design, since the feedback can lead to instability. However, Equation (8) is a simple linear combination of signals at different time and such a sequential logic equation can be implemented in hardware that performs calculations at a high sample rate.

### 3.3. Requirements and Hardware

The system to design is made of a first filter based on a QCM coupled with a second filter implemented in a hardware. These constraints require mainly two conditions to fulfill. Firstly, the sampling frequency $f_{s}$ of the hardware must be high enough when compared to the RtF $f_{r}$. The Nyquist condition demands $f_{s}>2f_{r}$, and a minimum of 10 samples per period is fixed here to describe each sine wave in the digital system. The minimum sampling frequency then satisfies $f_{s}=10f_{r}$. Given that the lowest RtF of commercial QCMs is between 1 MHz and 2 MHz, we can then set the highest RtF for which our design can work at 2 MHz, which thus requires a minimum sampling frequency of $f_{s}=20\;MHz$. Secondly, Equation (8) requires each addition and multiplication to be done within only a few time samples because of the IIR feedback. The different operations thus have to be carried out in a few nanoseconds only: massive parallel computation is then necessary.

A FPGA is a configurable integrated circuit allowing to carry out parallel calculations for combinational logic circuits and data storing (registers) for sequential logic circuits at a rate of several dozen of megahertz. The FPGA is then the hardware chosen here, and we specifically selected the Red Pitaya card to implement our design, since this board integrates all of the components needed for our application. Indeed, it includes two processor cores along with the FPGA (Zynq7000), two analog-to-digital converters (ADC) and two digital-to-analog converters (DAC) for communication with an analog system, a SD card slot, and an Ethernet connector. The clock signal of the DACs and ADCs, also used to synchronize the registers in the FPGA, is equal to 125 MHz, which satisfies our requirements.

### 3.4. Filter Model

**Figure 4:**
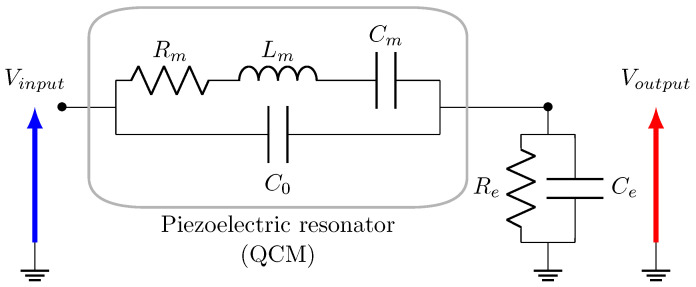
Low pass quartz cristal microbalance (QCM)-based resonant filter model.

The filter output must represent the resonator displacement or its equivalent the electrical charge in order to implement mode localization between two filters following Figure 1. From this consideration, a filter including a QCM based on the Butterworth-Van-Dyke model can be designed, by simply connecting one of its terminals to a capacitor $C_{e}$ in parallel of a resistor $R_{e}$, as depicted in Figure 4.

The transfer function H(s) of this electrical circuit is given by

(9) $$\left\{\begin{array}{cc} H(s) & =\frac{\left(1+\epsilon \right)\;\cdot \;s^{3}+\frac{1}{Q}\;\cdot \;s^{2}+\left(1+g_{1}\right)\;\cdot \;s}{\left(1+\epsilon \right)\;\cdot \;\left(1+g_{2}\right)\;\cdot \;s^{3}+\left[\frac{1+g_{2}}{Q}+g_{2}\;\cdot \;g_{3}\;\cdot \;\left(1+\epsilon \right)\right]\;\cdot \;s^{2}+\left[1+g_{1}+g_{2}+\frac{g_{2}\;\cdot \;g_{3}}{Q}\right]\;\cdot \;s+g_{2}\;\cdot \;g_{3}}\\ Q & =\frac{1}{R_{m}}\;\cdot \;\sqrt{\frac{L_{m}}{C_{m}}},\;\omega_{0}=\frac{1}{\sqrt{L_{m}\;\cdot \;C_{m}}},\;\epsilon =\frac{\delta L_{m}}{L_{m}},\;g_{1}=\frac{C_{m}}{C_{0}},\;g_{2}=\frac{C_{e}}{C_{0}},\;g_{3}=\frac{\omega_{e}}{\omega_{0}},\;\omega_{e}=\frac{1}{R_{e}\;\cdot \;C_{e}}\\ s & =j\;\cdot \;\frac{\omega }{\omega_{0}} \end{array}\right.$$

The parameter $g_{1}$ only depends on the QCM, and $g_{2}$, $g_{3}$ must be chosen. In particular, $g_{3}$ must satisfy $g_{3}\ll 1$ in order to obtain an integrator behavior of the output impedance, then almost equivalent to a single capacitor. Indeed, the output impedance, at the angular frequency $\omega_{0}$, equals:

(10) $$\begin{array}{cc} Z_{e}\left(\omega_{0}\right) & =\frac{R_{e}}{1+\frac{j}{g_{3}}} \end{array}$$

### 3.5. Digital Filter Implementation

**Figure 5:**
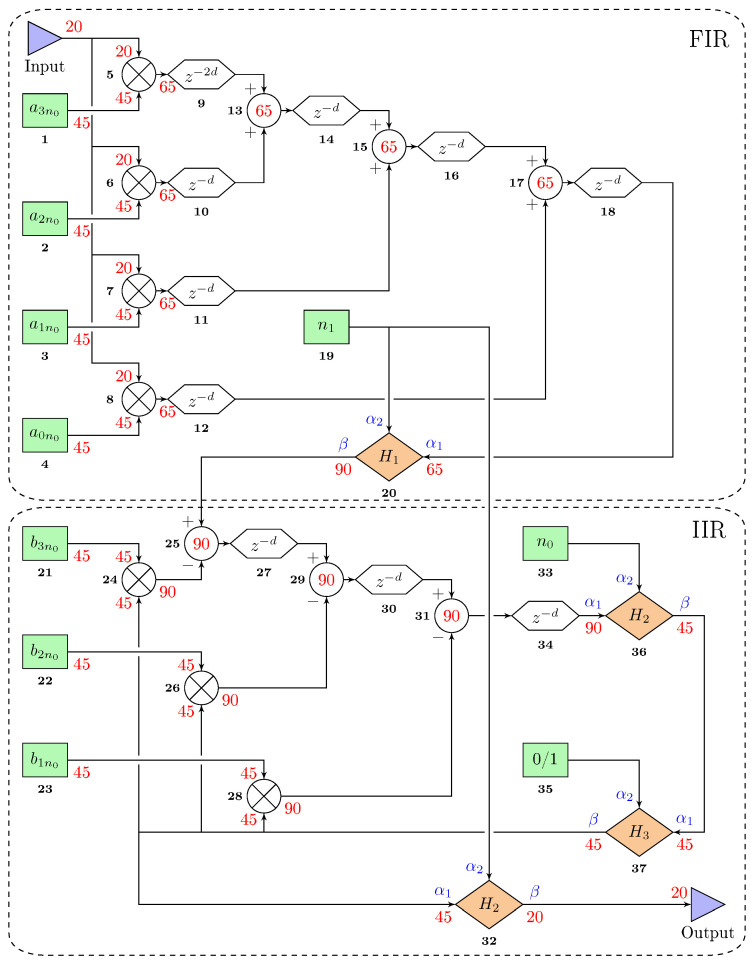
Simplified diagram of the implemented digital filter in the field programmable gate array (FPGA) and representing resonator 1 in Figure 1. The black and bold numbers are the blocks identifiers. The numbers in red correspond to the number of bits on which the numbers are encoded and *d* is the decimation factor. The hexagonal blocks are registers, the circular ones with a cross inside are multipliers, the green blocks are tunable values, and the orange ones are custom sources detailed in Table 1.

**Figure 6:**
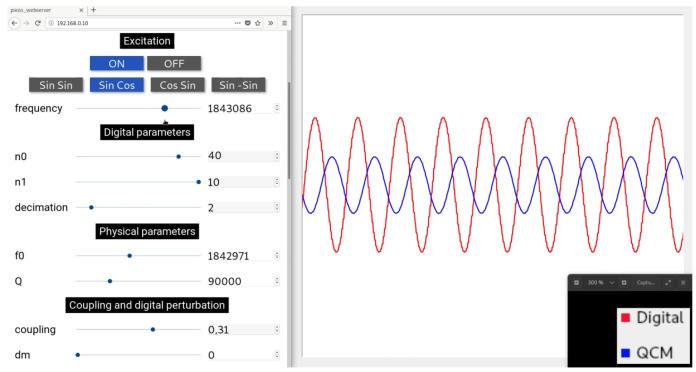
Graphical user interface (GUI) screenshot: Webserver on the left (buttons, sliders, and spin boxes for parameters tuning) and GNU radio on the right (numerical oscilloscope).

Replacing Equation (6) in Equation (9) yields an expression in the form of Equations (7) and (8). Figure 5 depicts its implementation in the FPGA. The entire design was done under Vivado design suite 2019.1 and a Python 3 GUI has been programmed for the control of the filter parameters as well as data recording. Figure 6 illustrates this GUI.

Equation (8) requires strict timing constraints that may not be met by the hardware, especially in the IIR part. It can be seen that a multiplication and an addition must be done during the same time sample (blocks 28–31, 26–29, and 24–25), which the FPGA cannot do experimentally. Therefore, an additional Verilog source has been set up in order to proceed to a down-sampling based on the decimation factor *d* (natural number). The new sampling frequency $f_{d}$ then follows Equation (11).

(11) $$f_{d}=\frac{f_{s}}{d}$$

This new clock is applied to each block of the filter to the other blocks of the design. Experimentally, the lowest value of *d* for which the timing constraint is respected is d=2, regardless of the number of digits on which the numbers are coded due to the parallel computation.

Appendix B provides more details on the digital filter design.

### 3.6. Fabrication of the QCM Based Filter

Now that the digital filter is set up, the model from Figure 4 needs to be implemented with analog components, namely a QCM, a capacitor, and a resistor. First of all, it is necessary to carry out an impedance matching. Indeed, the input signal of the filter corresponds to the output of the Red Pitaya DAC. Since this output is designed to supply circuits with an impedance equal to 50 Ω, it is necessary to add a 50 Ω$R_{load}$ resistor in parallel before the QCM. In order to ensure that the impedance of the rest of the circuit is constant and sufficiently high compared with $R_{load}$, a first voltage follower $OA_{1}$ is set up between $R_{load}$ and the QCM. Because the output amplitude may not exceed 1V because of the ADC voltage range, it is necessary to add a voltage divider stage between the output of the QCM and the ADC, which is the role of $R_{1}\in \left[0\ldots 2\;k\Omega \right]$ and $R_{2}=1\;k\Omega$, a sufficiently low impedance as compared to that of the ADC (1 MΩ). Once more, a voltage follower $OA_{2}$ is added to ensure high impedance at the QCM output, so as not to disrupt the behavior of the filter. Finally, a resistor $R_{0}$ of 50 Ω is connected before the QCM input to avoid experimental high-frequency parasitic oscillations between the two operational amplifiers which have a high slew rate. The operational amplifier chosen for our application is the OA LT1358 from Linear Technology, because of its slew rate and gain-bandwidth. Indeed, we are working with 2 MHz RtF resonators having a gain around 10 only at the resonance because of the feedthrough transmission (parallel capacitance of the QCM electrical model).

**Figure 7:**
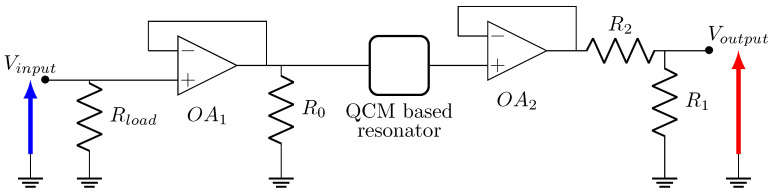
Detailed circuit of the entire analog filter standing for resonator 2 in Figure 1. The QCM based resonator corresponds to Figure 4.

**Figure 8:**
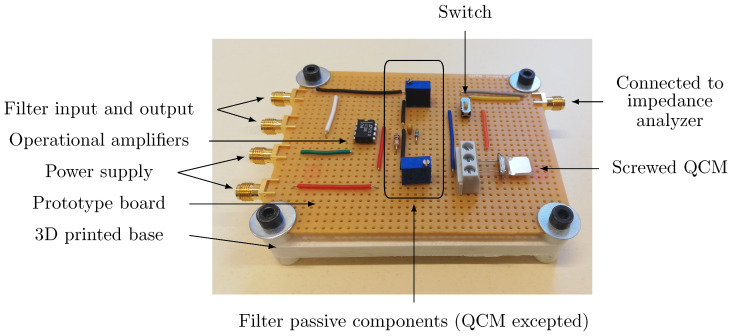
Picture of the fabricated QCM based resonator corresponding to Figure 7. The QCM is set horizontally in order to facilitate further mass deposition on its surface through a liquid drop deposition, and a switch has been added to enable the QCM to be connected either to the rest of the filter or to an impedance analyzer. The QCM can be easily changed, since it is fixed with simple screws.

The chosen QCM is a simple quartz resonator of RtF 1.8 MHz and its packaging is removed in order to access the surface of the quartz. Its electrical characteristics are measured by the mean of an impedance analyzer E4990A from Keysight, so as to calculate the different parameters from Equation (9). In particular, its Q-factor equals to 115,000. Concerning the output impedance, the condition $g_{3}\ll 1$ must be satisfied while avoiding additional unwanted behavior. For instance, high values of $C_{e}$ will lead to very low output amplitudes, and low values of $C_{e}$ will induce high output amplitudes and, thus, a saturation of the Red Pitaya’s ADC voltage. As a consequence, the chosen values are $R_{e}=100\;k\Omega$ and $C_{e}=100\;pF$. The fabricated electrical circuit including the QCM is depicted in Figure 7 and Figure 8.

The different components of the QCM based filter have been hand-soldered on a prototype board, which is screwed onto a 3D printed base. SMA connectors are used to connect the device to the rest of the system.

### 3.7. Implementation of the Coupled System

**Figure 9:**
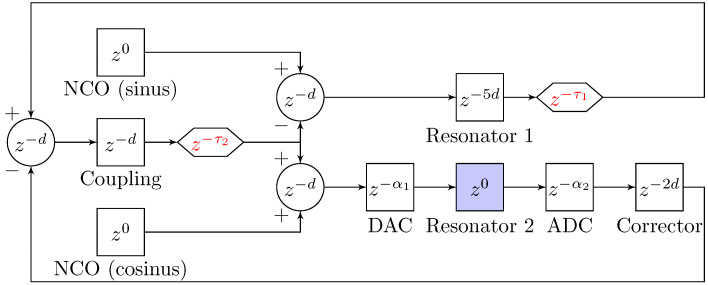
Global sketch of the coupled system, including the delays corresponding to each operation. The only non digital element is resonator 2 (QCM based filter), in blue. Tunable delays were added for timing compensations, in red.

Figure 9 depicts a sketch of the entire system.

The numerically controlled oscillator (NCO) generates a sinus or a cosinus signal on 14 bits with a tunable frequency. A sinus from the NCO is chosen to be the reference for the phase and, therefore, for the delays. Each mathematical operation (addition or multiplication) requires one register that releases data every *d* samples. The corrector contains both an addition and a multiplication since its role is to multiply resonator 2 output by the inverse gain of the voltage divider $R_{1}$ and $R_{2}$ from Figure 7, and it also compensates any potential offset with an addition. The DAC and ADC have a delay of a few dozen of nanoseconds and are denoted $\alpha_{1}$ and $\alpha_{2}$, respectively. Resonator 1 introduces a delay of 5.d, which corresponds to the delay between the input and output signals that can be counted in Figure 5, and resonator 2 is considered not to add any delay. Two additional tunable registers were added to balance these delays. Indeed, $\tau_{1}$ enables both resonators to be in phase, and $\tau_{2}$ ensures the coupling contribution is in phase with the resonators output on the next period. Without this last tuning, the second mode amplitude is greater than it should be, thus leading to a ADC and DAC saturation.

**Figure 10:**
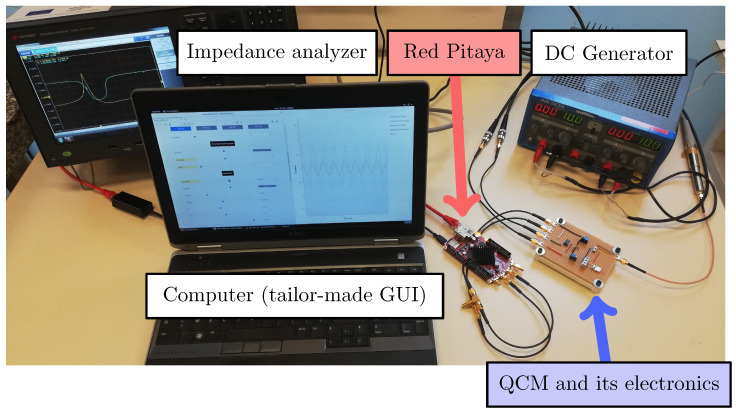
Experimental setup including the piezoelectric resonator, a DC generator, a computer and its GUI, the Red Pitaya, and an impedance analyzer E4990A from Keysight that enables to measure the QCM RtF after each mass deposition for further comparison with ML based methods. The DC generator supplies the operational amplifiers.

A picture of the experimental setup from Figure 9 is given in Figure 10. It allows the experimental demonstration of ML, as presented in the following section.

## 4. Results

### 4.1. Description of the Experiments

Before the implementation of ML in the hybrid system, the digital resonator parameters must be adjusted, so that both resonators responses are identical, using the following simplified protocol:

1. No coupling is applied.
2. The output resistance $R_{1}$ must be tuned to set the resonance amplitude to less than 1V (limit imposed by the ADC of the Red Pitaya).
3. The values of the different parameters are entered in the FPGA.
4. Both excitation signals are set in phase.
5. $f_{0}$ (digital filter) is tuned such as the resonances of the two filters experimentally match.
6. The corrector gain is tuned: it makes possible to compensate the voltage divider but also to experimentally adjust the resonance amplitude of the QCM-based filter to that of the digital filter.
7. Tuning the digital Q-factor enables the bandwidth of the two filters to be experimentally identical.
8. $\tau_{1}$ is modified so that the two uncoupled resonators are experimentally in phase and $\tau_{2}$ must be adapted to this value according to the relation $\tau_{1}+\tau_{2}+8d=\frac{f_{s}}{f_{r}}$.
9. The two excitation signals are set with a phase of $\frac{\pi }{2}$ rad in order to observe both modes.
10. The coupling value is eventually tuned to fit the best configuration in terms of sensitivity.

**Figure 11:**
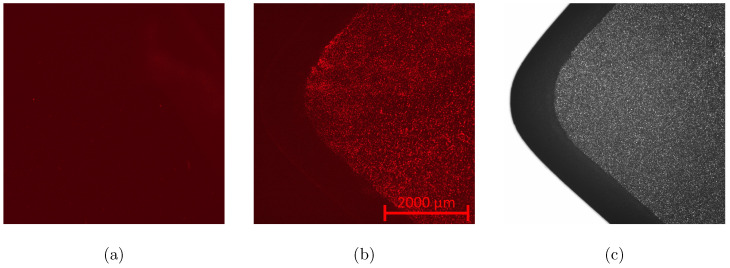
Surface of the QCM electrode before and after a single deposition of around $3\times 10^{5}$ fluorescent particles. Images taken with the microscope Axio from Zeiss and a magnification of 2.5. (**a**): Before deposition and under red lightning (**b**): After deposition and under red lightning (**c**): After deposition and without red lightning.

The experiment consists in the deposition of micro particles at the surface of the QCM. After each deposition, frequency responses are measured over a frequency range containing both modes. The change in the resonant frequency of the QCM alone is also measured, which will be used for the calculation of the added masses thanks to the normalized sensitivity of the RfF of one half. Each NS value is then calculated as the relative amplitude shift over the relative mass shift for each deposition of particles. The particles used in these experiments are fluorescent melamine resin particles MF-NB-COOH-S1058 from microparticles GmbH, Berlin. They have a diameter of 920 nm, a density of 1.510, and are put in an ethanol solution for its high wetability and evaporation rate. The volume of the drop is fixed at 1 μ L, because such a drop experimentally spreads all over the electrode without overflowing the edge of the QCM, as visible in Figure 11.

In order to demonstrate ML, we wish here that the sensor operates within the sensitivity range that is up to a normalized perturbation $\epsilon =\frac{\delta m}{m}=\frac{2}{Q}$, as shown by the theoretical results previously presented. With our Q-factor of 115,000, this limit can roughly be set around ϵ=20 ppm. In order to stay in the sensitive range, a maximum value of $\epsilon_{f}=15\;ppm$ is chosen. The effective mass *m* of the QCM must now be estimated to calculate the mass perturbation $\delta m_{f}$ corresponding to $\epsilon_{f}$, knowing that $\epsilon_{f}=\frac{\delta m_{f}}{m}$. This effective mass *m* can be calculated using the Sauerbrey equation and the RtF sensitivity to mass perturbation, as written in Equation (12).

(12) $$\left\{\begin{array}{cc} \delta m & =-\frac{A\;\cdot \;\sqrt{\rho_{q}\;\cdot \;\mu_{q}}}{2f_{r}^{2}}\;\cdot \;\delta f\\ \frac{\delta f_{r}}{f_{r}} & =-\frac{1}{2}\;\cdot \;\frac{\delta m}{m} \end{array}\right.$$

where *A*, $\rho_{q}$, $\mu_{q}$, and $f_{r}$ are the electrodes area, density, shear modulus of quartz and resonant frequency, respectively.

The combination of these equations leads to Equation (13), which is the effective mass expression.

(13) $$\begin{array}{cc} m & =\frac{A\;\cdot \;\sqrt{\rho_{q}\;\cdot \;\mu_{q}}}{4f_{r}} \end{array}$$

In our case, the electrode is a square of side 7.3 mm, $f_{r}=1.843\;MHz$, $\rho_{q}=2648\;kg\;m^{-3}$ and $\mu_{q}=2.947\times 10^{10}\;kg\;m^{-1}\;s^{-2}$, which leads to

(14) $$\begin{array}{cc} m & =64.0\;mg \end{array}$$

As a consequence, $\delta m_{f}=1\;\mu g$. For a proper demonstration of ML, five consecutive mass depositions are carried out, requiring δm=200 ng to be dropped each time, which approximately corresponds to $3\times 10^{5}$ particles. The available solution has therefore been diluted to reach this amount of particles per volume of 1 μL.

The vibration amplitudes are calculated as the average of peak-to-peak amplitude values over several periods: each of this vibration amplitude is obtained from four uncorrelated data set from the FPGA of 2048 time samples, which roughly correspond to 118 periods at a frequency of 1.8 MHz.

### 4.2. Experimental Results

Five mass depositions have been carried out at the QCM surface in order to demonstrate ML in our hybrid system using the tracked resonance or fixed frequency methods. The system is tuned to achieve a high sensitivity before the experiment, which was performed four times. The applied coupling stiffness equals κ=0.15 in order to avoid mode aliasing on both resonators. This coupling value is much higher than those that are shown on the maps in Figure 2 and Figure 3. This is due to the fact that with the transfer function from Equation (9), mode aliasing occurs for higher coupling values because the frequency of the second mode is located between series and parallel resonances. However, the above-mentioned properties on sensitivities and sensitive ranges are conserved with this system.

**Figure 12:**
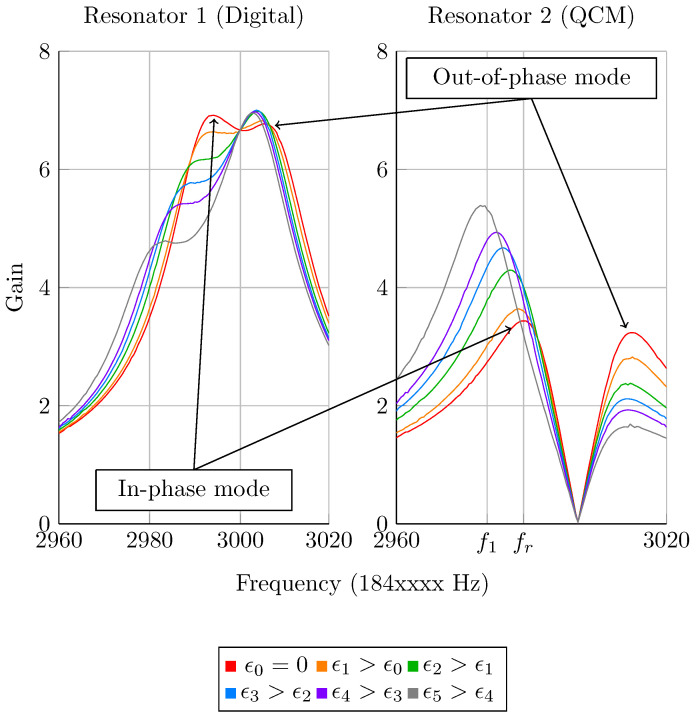
Experimental amplitude Bode diagrams of the coupled system digital-QCM for a coupling ratio κ=0.15 and different mass perturbations $\epsilon_{i}$ applied on resonator 2, which is excited with a phase of $\frac{\pi }{2}$ rad.

Figure 12 depicts the amplitude Bode diagrams of such an experiment. It is observed that the first mode localizes again on the resonator on which the mass perturbation is introduced and that mode aliasing almost occurs on resonator 1, which is not the case of resonator 2, because of the anti-resonance generated by the excitation phase. The proximity of both resonances and this anti-resonance is also the cause of the lower amplitude on resonator 2. These phenomena due to the phase of 90 degrees between both excitation are inverted when its sign is changed. The resonant frequency of the first mode for the second resonator, in the balanced configuration (red curve), $f_{r}$, enables calculating $f_{1}$, also depicted in Figure 12.

**Figure 13:**
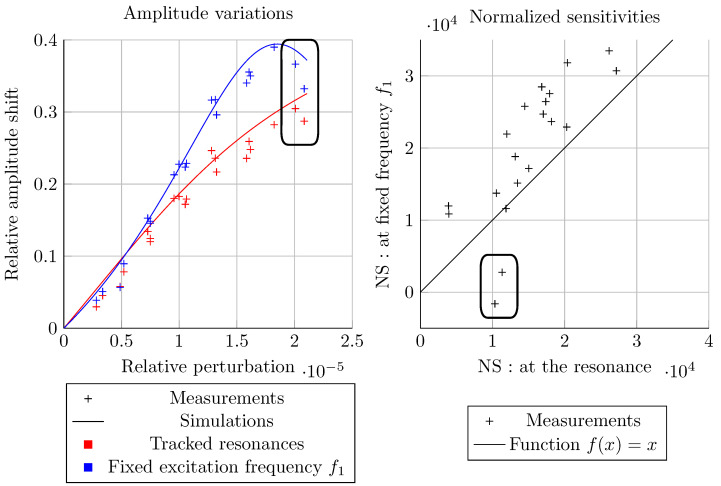
Amplitude variations and their corresponding normalized sensitivities of the first mode of resonator 2 where the particles are deposited for both methods: tracked resonances and fixed excitation frequency $f_{1}$.

The relative amplitude shifts of the four experiments are plotted with respect to the relative perturbation $\epsilon =\frac{\delta m}{m}$ in Figure 13, along with the calculated NS for both methods, based on mode 1 of resonator 2. It is first observed that the maximum perturbation is around 20 ppm as chosen previously. Moreover, both experimental and theoretical data are close, showing the successful implementation of ML. The small drifts observed are most likely due to measurement noise and a slight mistuning of the system. We also observe that both of the methods are nonlinear, and that the fixed frequency one yields higher NS, up to a value above $3\times 10^{4}$. With our Q-factor, the maximum theoretically reachable NS with this method is around $4.4\times 10^{4}$. However, this value is not reached here, because the coupling value has been chosen in order to avoid mode aliasing: it must be slightly lower to enhance the NS. Indeed, as visible in Figure 2 and Figure 3, mode aliasing occurs on resonator 1 for the optimum configuration. The NS value drops by half, from around $3\times 10^{4}$ down to $1.5\times 10^{4}$ for both methods, as predicted. However, two NS values surrounded in black on both graphs are drastically lower for the fixed frequency method, which corresponds to the two highest perturbations: for $\epsilon =\frac{2}{Q}$, the resonance is reached and the NS drops down to zero at the fixed excitation frequency $f_{1}$.

Finally, this new method yields sensitivities slightly higher than the classic amplitude shift at the resonance while avoiding the design of a closed loop or the need to proceed to time consuming frequency sweeps. Its dynamic range is limited to $\frac{2}{Q}$, which is not the case when the resonances are followed. However, both of these methods have a similar sensitive range (still $\frac{2}{Q}$), which is not a limitation when it comes to lower the limit of detection (LoD).

## 5. Discussion

The configurations for which the mass NS is maximum for a given pair of weakly coupled resonators in terms of coupling value and Q-factor can be summarized, as follows. The maximum NS is proportional to the Q-factor and inversely proportional to the coupling ratio κ, until mode aliasing occurs. Furthermore, the sensitive range (here fixed when the NS drops by half) is also inversely proportional to the mass mismatch ϵ. This sensitive range equals $\frac{2}{Q}$ when the resonance is tracked, but also when the system is excited at a fixed frequency $f_{1}=f_{r}\;\cdot \;\left(1-\frac{1}{2Q}\right)$. It is also demonstrated that this method yields sensitivities that are slightly higher than the classic amplitude shift at the resonance, because the signal increase is due to both the RtF downshift and mode localization. This information shows that mode localized sensors can operate at a fixed excitation frequency in open loop, which avoid either a time consuming frequency sweep or the implementation of a positive feedback to follow the resonance.

We implemented both methods experimentally in order to confirm these theoretical results. To do so, we designed a new type of mode localized sensor based on a hybrid system enables to get rid of any geometric constraints for the implementation of the coupling and make it possible to finely tune the different resonator parameters. This way, mode localization can be efficiently implemented on shear waves resonators yielding high Q-factors and, thus, high sensitivities. The experimental amplitude shifts and corresponding sensitivities validate the theoretical results and our sensitivities are among the highest in the mode localized sensors found in the literature, as shown in Table 2.

In summary, the main performances unique to our system can also be listed below. Indeed, our system enables to:

- Generate a second mode of vibration and exploit mode localization on a shear wave resonator widely used in bio-sensing (QCM) with high Q-factor (up to at least 200,000) and high resonance frequency (up to at least 2 MHz).
- Carry out a complete tuning of the digital filter parameters and the coupling value before each experiment. This allows to reach high NS values compared with the literature (up to $3\times 10^{4}$) and to get rid of any initial imbalance between the resonators before the measurements.
- Replace the QCM easily and adapt to the geometry of the piezoelectric resonator if needed.
- Exploit the mode localization phenomenon without tracking the resonances by exciting the system at a fixed frequency $f_{1}=f_{r}\;\cdot \;\left(1-\frac{1}{2Q}\right)$.

If the performances of our system are satisfying in regard to the chosen figure of merit (normalized sensitivity), our system in its current state still has limits. For instance, one advantage of ML is common mode rejection, but the digital nature of one resonator prevents this phenomenon happening. Indeed, any change in the ambient temperature or pressure affects only the QCM and not the digital filter, thus leading to an imbalance, the localization of energy and a misinterpretation of the measurements. Such an imbalance was however not observed in the time frame of the experiments. Even though our system does not benefit from mode rejection, it is reminded that the system can be balanced before each mass deposition, thus guaranteeing high and known sensitivity by the cancel of any long term drift. However, a study on the temperature sensitivity should be carried out in a future work in order to evaluate whether a temperature controlled environment is needed or not for this sensor.

Many improvements are possible on the presented hybrid system. For instance, the literature shows that an array of resonators with less stiff external resonators yields higher NS for a given coupling value κ. The resolution of the eigenvalue problem in the case of a 3 DoF system shows indeed that the two first modes get closer in frequency for a given value of κ (compared with a 2 DoF system), which increases the NS. Nonetheless, one should also keep in mind that mode aliasing might occur for higher values of κ, which could thus prevent from reaching such high NS. As a first perspective of this work, extended studies could be carried out to find out the actual gain in sensitivity and decrease in LoD of such systems. If the decrease in LoD can be proved with a 3 DoF system with stiffer middle resonator, a second digital filter could be implemented in the FPGA, allowing to reproduce and exploit this configuration with our fixed frequency method.

Another possible development concerns the way to calculate the vibration amplitudes. Instead of averaging the peak to peak values over many periods, it could be considered to average the amplitudes of the Fourier transforms at the excitation frequency over several acquisitions, which should be more accurate since the noise from the other frequencies is not taken into account in this calculation. In addition, it could be considered to apply specific windows on the recorded signals such as the Hanning window, in order to limit the spectral leakage around the resonant frequency. More generally, the different noise sources that corrupt the signals should be identified and analyzed in order to find a way to decrease the LoD quite high so far (quantization noise, operational amplifiers noise, clock jitter and so on).

Furthermore, the Matlab^®^simulations given in Figure 13 could provide calibration data in order to calculate the mass mismatch using mode localization and not the frequency shift, which is the primary purpose of the sensor. This calibration could even include a correction related to the change in Q-factor caused by the particles depositions, since the NS is directly proportional to it, even though no significant Q-factor drift were observed during the experiments.

Finally for time saving, the tuning protocol could be automatized. It is so far executed by an operator, but the digital aspect of the system could accommodate an additional routine that would automate this delicate part of the process either when the operator needs to apply it, or to guarantee high sensitivity on a larger range by an automatic downshift of the digital filter resonant frequency as the perturbation increases.

## 6. Conclusions

This paper presents a prototype of mass sensor based on mode localization in a hybrid system made of a quartz resonator and a FPGA that yield higher sensitivities than those found in the literature. The digital aspect enables to reach optimal conditions in term of sensitivity with a fine tuning of different parameters such as the RtF or the coupling stiffness, and to implement a coupled structure including shear wave resonators that have high Q-factors. Furthermore, we show both theoretically and experimentally that the sensitive ranges are similar between two distinct excitation methods in open loop: a frequency sweep over both resonances allowing to measure the amplitudes at the resonances on the one hand, and a fixed excitation frequency $f_{1}=f_{r}\;\cdot \;\left(1-\frac{1}{2Q}\right)$ at which the vibration amplitudes are measured on the other hand. The second one however yields higher sensitivities than the first one, and their maximum sensitivities are 0.35×Q and 0.25×Q, respectively. These results pave the way for a new generation of low LoD resonant mass sensors without resonance tracking, which results in a gain of time in an open loop configuration.

## Figures and Tables

**Table 1:** Expressions of the transfer functions from Figure 5.

Source	$H_{1}$	$H_{2}$	$H_{3}$
**Expression**	$\beta =\alpha_{1}\;\cdot \;2^{\alpha_{2}}$	$\beta =\frac{\alpha_{1}}{2^{\alpha_{2}}}$	$\beta =\alpha_{1}$, for $\alpha_{2}=1$β=0 otherwise
**Implementation**	Left bit-shifting	Right bit-shifting	Conditional loop
**Number of required registers**	0	0	0

**Table 2:** Comparison of our hybrid sensor with a few devices using mode localization developed in different teams. Both a previous published work on a QCM of 1 MHz RtF and those from this manuscript are presented here.

Parameter	Literature	This Work
$f_{0}$ (Hz)	$1.34\times 10^{4}$ [4], $1.49\times 10^{4}$ [3]	$1.84\times 10^{6}$
	$3.11\times 10^{5}$ [24]	$1.00\times 10^{6}$ [21]
*Q*	^1^$1.34\times 10^{2}$ [4],^2^ $6.22\times 10^{3}$ [3]	$1.15\times 10^{5}$
	^2^$2.12\times 10^{4}$ [24]	^2^$1.70\times 10^{5}$ [21]
Maximum normalized sensitivity reached		
2 DoF	$4.00\times 10^{2}$ [4], ^3^ $2.34\times 10^{2}$ [24]	$3.00\times 10^{4}$, $3.50\times 10^{4}$ [21]
3 DoF	^4^$1.36\times 10^{4}$ [3]	future work

^1^ calculated from the bandwidth, ^2^ in vacuum, ^3^ calculated, knowing the normalized sensitivity of frequency shift is $\frac{1}{2}$, ^4^ amplitude ratios as sensor output.

## Abbreviations

The following abbreviations are used in this manuscript:

ADC	Analog to Digital Converter
DAC	Digital to Analog Converter
DoF	Degree of Freedom
FIR	Finite Impulse Response filter
FPGA	Field Programmable Gate Array
GUI	Graphical User Interface
IIR	Infinite Impulse Response filter
LoD	Limit of Detection
ML	Mode Localization
NS	Normalized Sensitivity
QCM	Quartz Cristal Microbalance
RtF	Resonant Frequency

## Appendix A. Proofs of Properties 1 and 2

The proof of Property A1 is given in [24].

**Property** **A1.**
*Expressions of the eigenvectors shifts due to a small mass variation in a 2 DoF coupled array made of undamped and initially identical resonators.*

(A1) $$\left\{\begin{array}{cc} \delta \omega_{n} & ≃-\frac{\delta \mu_{n,n}}{2}\;\cdot \;\omega_{0n}\\ \delta u_{n} & ≃-\frac{\delta \mu_{n,n}}{2}\;\cdot \;u_{0n}+\frac{\delta \mu_{p,n}}{{\left(\frac{\omega_{0p}}{\omega_{0n}}\right)}^{2}-1}\;\cdot \;u_{0p}\\ \delta \mu_{i,n} & =u_{0i}^{T}\;\cdot \;\delta M\;\cdot \;u_{0n}\\ n,p & \in \{1,2\},\;p\neq n \end{array}\right.$$

*where $u_{0n}$, $\delta u_{n}$ and $\delta \omega_{n}$ are the nth eigenvector before the addition of mass, the small variation of this eigenvector and the corresponding eigenfrequency shift after the introduction of a mass perturbation in the system, respectively. δM is the diagonal two by two matrix containing the normalized small mass shifts $\frac{\delta m}{m}$ where m is the mass of each resonator and $\omega_{0n}$ is the $n^{th}$ eigenfrequency.*

**Proof of Property** **1.** Considering two identical and coupled undamped resonators of stiffness *k*, mass *m*, coupling stiffness $k_{c}$ and with the notations from Property A1, we can write:

(A2) $$u_{01}=\frac{1}{\sqrt{2}}\;\cdot \;\left(\begin{array}{c} 1\\ 1 \end{array}\right),\;u_{02}=\frac{1}{\sqrt{2}}\;\cdot \;\left(\begin{array}{c} 1\\ -1 \end{array}\right),\;\omega_{01}=\sqrt{\frac{k}{m}},\;\omega_{02}=\sqrt{\frac{k+2k_{c}}{m}}$$

Assuming a mass perturbation $\epsilon =\frac{\delta m}{m}$ occurs on resonator 1, we have:

(A3) $$\delta \mu_{1,1}=\delta \mu_{2,1}=\frac{\epsilon }{2}$$

Assuming weak coupling, the frequency gap between the two modes shrink and the influence of the first eigen vector on its own variation becomes negligible. Therefore, the variation of the first eigenvector is:

(A4) $$\begin{array}{cc} \delta u_{1} & ≃\frac{1}{2}\;\cdot \;\frac{\epsilon }{{\left(\frac{\omega_{02}}{\omega_{01}}\right)}^{2}-1}\;\cdot \;u_{02} \end{array}$$

Equations (A2) and (A4) give the famous result on low coupling NS [2]:

(A5) $$\begin{array}{c} ns=\frac{|\delta u_{1}|}{\epsilon }=\frac{1}{4\kappa } \end{array}$$

where $\kappa =\frac{k_{c}}{k}$. However this result does not take mode aliasing into consideration, where the two modes merge because of the resonance bandwidth coming for internal losses. Assuming that damped resonators have a frequency bandwidth $\Delta \omega_{-3dB}$, we here set an anti-aliasing condition such as [3]:

(A6) $$\omega_{02}-\omega_{01}>\Delta \omega_{-3dB}$$

Considering the the Q-factor can be expressed as follow:

(A7) $$Q=\frac{\omega_{01}}{\Delta \omega_{-3dB}}$$

The anti-aliasing condition becomes:

(A8) $${\left(\frac{\omega_{02}}{\omega_{01}}\right)}^{2}>{\left(1+\frac{1}{Q}\right)}^{2}$$

Considering high Q-factors, we only keep the first order. The minimum anti-aliasing condition is then defined below:

(A9) $$\begin{array}{cc} {\left(\frac{\omega_{02}}{\omega_{01}}\right)}^{2}-1 & ≃\frac{2}{Q} \end{array}$$

Replacing Equation (A9) in Equation (A4) yields:

(A10) $$\begin{array}{c} \delta u_{1}≃\frac{Q\;\cdot \;\epsilon }{4}\;\cdot \;u_{02} \end{array}$$

Therefore:

(A11) $$\begin{array}{c} ns_{1}=\frac{|\delta u_{1}|}{\epsilon }≃\frac{Q}{4} \end{array}$$

□

**Proof of Property** **2.** Assuming that any signal amplitude variation at fixed excitation frequency corresponds almost only to the resonance shift towards lower frequencies, there is at least one excitation frequency at which the sensitivity to the mass perturbation is maximum. We aim at finding this frequency along with the value of the maximum NS. The transfer function displacement over excitation force of a damped resonator with a slight mass perturbation $\epsilon =\frac{\delta m}{m}\ll 1$ is:

(A12) $$H\left(s\right)=\frac{1}{\left(1+\epsilon \right)\;\cdot \;s^{2}+\frac{1}{Q}\;\cdot \;s+1}$$

where $s=j\;\cdot \;\frac{\omega }{\omega_{0}}$, ω the excitation angular frequency and $\omega_{0}$ the resonant angular frequency. Let’s excite the system at its RtF and define X as:

(A13) $$\begin{array}{cc} X & =\left|H(s=j)\right|\\ X & =\frac{Q}{\sqrt{1+\epsilon^{2}\;\cdot \;Q^{2}}} \end{array}$$

The NS is then written as:

(A14) $$\begin{array}{cc} ns & =\frac{\partial X}{\partial \epsilon \;\cdot \;X_{\epsilon =0}}\\ ns & =\frac{-Q^{2}\;\cdot \;\epsilon }{{\left(1+\epsilon^{2}\;\cdot \;Q^{2}\right)}^{\frac{3}{2}}} \end{array}$$

We now aim at finding the maximum absolute value of this function. ns is a negative function of ϵ which equals 0 when ϵ equals 0, and that tends to 0 when ϵ tends to infinity. Therefore, ns has a maximum absolute value we wish to find. To this purpose, let’s now derive ns with respect to ϵ:

(A15) $$\begin{array}{cc} \frac{\partial ns}{\partial \epsilon } & =\frac{-Q^{2}\;\cdot \;{\left(1+\epsilon^{2}\;\cdot \;Q^{2}\right)}^{\frac{3}{2}}+Q^{2}\;\cdot \;\epsilon \;\cdot \;\left(3Q^{2}\;\cdot \;\epsilon \sqrt{1+\epsilon^{2}\;\cdot \;Q^{2}}\right)}{{\left(1+\epsilon^{2}\;\cdot \;Q^{2}\right)}^{3}} \end{array}$$

We now solve the equation

(A16) $$\begin{array}{cc} \frac{\partial ns}{\partial \epsilon }=0 & \Leftrightarrow -Q^{2}\;\cdot \;{\left(1+\epsilon^{2}\;\cdot \;Q^{2}\right)}^{\frac{3}{2}}+Q^{2}\;\cdot \;\epsilon \;\cdot \;\left(3Q^{2}\;\cdot \;\epsilon \sqrt{1+\epsilon^{2}\;\cdot \;Q^{2}}\right)=0\\ \frac{\partial ns_{0}}{\partial \epsilon }=0 & \Leftrightarrow \epsilon =\frac{1}{\sqrt{2}Q} \end{array}$$

We then obtain an estimation of the maximum absolute value of the NS, using Equation (A14):

(A17) $$\begin{array}{cc} ns_{max} & ≃0.38\times Q \end{array}$$

The amplitude variation is here mainly due to the RtF downshift. Assuming the frequency response in amplitude is symmetric with the RtF, there are two frequencies at which the system can be excited to obtain this maximum NS, above and below the RtF. Therefore, we can calculate one of these two frequencies using the frequency NS of one half, as follow:

(A18) $$\begin{array}{c} \frac{f_{1}-f_{r}}{f_{r}}\;\cdot \;\frac{1}{\epsilon }=-\frac{1}{2} \end{array}$$

where $f_{r}$ and $f_{1}$ are the RtF before and after mass deposition, respectively. In order to obtain a sensitive range slightly higher, we rather choose ϵ=1/Q, for which the NS is still

(A19) $$\begin{array}{c} ns_{2}≃0.35\times Q \end{array}$$

We then have:

(A20) $$\begin{array}{cc} f_{1} & =f_{r}\;\cdot \;\left(1-\frac{1}{2Q}\right) \end{array}$$

□

## Appendix B. Digital Filter Details

This appendix provides additional details on the digital filter implemented in the FPGA. It mainly refers to Figure 5.

### Appendix B.1. Feedback Switch

When the filter coefficients are modified throughout the GUI (filter tuning), values of no physical significance can appear and propagate in the closed loop of the IIR, thus generating unpredictable output. This phenomenon last only a fraction of second before the correct steady state is reached. However, the output value may reach high values, usually triggering overflow. Overflow, once introduced in the closed loop, has no chance to stabilize since the meaningless numbers do not only appear when the coefficients are modified, but propagates at each time sample and forever. For this reason, an automatically controlled switch (block 37) has been added on the output feedback: when the coefficient values are modified, the switch opens, sends a zero feedback and closes after one millisecond, a time large enough to allow a few thousand samples to pass through (it must be above 3×d, which corresponds to the blocks 27, 30 and 34), and small enough not to be a nuisance to the experiment. In this way, parametric instability can be suppressed when the coefficients are changed.

### Appendix B.2. Precision of the Filter Coefficients

Another problem with the digital filter is the stability of the IIR. Indeed the roots of the following function must have a modulus less than one.

(A21) $$\begin{array}{cc} H(z) & =z^{3}+b_{1}\;\cdot \;z^{2}+b_{2}\;\cdot \;z+b_{3} \end{array}$$

For a given set of parameters having a physical meaning such as Q>0, the roots of Equation (A21) always have their modulus smaller than one. However, the numbers implemented in the FPGA are only integers. Therefore, there is a need to multiply the coefficients $b_{1}$, $b_{2}$ and $b_{3}$ by a large integer in order to minimize the approximations done over these coefficients when injected in the hardware. Thus, these coefficients are multiplied by a power of two (parameter $n_{0}$) which will facilitate the inverse operation within the FPGA using right bit-shifting. This common method is called floating point method.

The roots of the following function have then been computed using Matlab^®^

(A22) $$\begin{array}{cc} H(z) & =2^{n_{0}}\;\cdot \;z^{3}+floor\left(2^{n_{0}}\;\cdot \;b_{1}\right)\;\cdot \;z^{2}+floor\left(2^{n_{0}}\;\cdot \;b_{2}\right)\;\cdot \;z+floor\left(2^{n_{0}}\;\cdot \;b_{3}\right) \end{array}$$

where floor(x) is the function that gives the greatest integer less than or equal to *x*. It can be shown through this simulation that the stability condition is fulfilled when $n_{0}>22$. This result is experimentally confirmed with the digital filter in the FPGA.

The coefficients $a_{i}$ and $b_{i}$ introduced in the FPGA are then called $a_{in_{0}}$ and $b_{in_{0}}$, with

(A23) $$\begin{array}{cc} a_{0n_{0}} & =floor\left(2^{n_{0}}\;\cdot \;a_{0}\right),\;a_{in_{0}}=floor\left(2^{n_{0}}\;\cdot \;a_{i}\right),\;b_{in_{0}}=floor\left(2^{n_{0}}\;\cdot \;b_{i}\right),\;i\in \left\{1..3\right\} \end{array}$$

The IIR part of the filter (Figure 5) must include a division by $2^{n_{0}}$ before the feedback loop in order to get back to the real value of the output, which is the role of the block 36.

Even though the digital filter is stable when $n_{0}>22$, its frequency response could not be the expected one because of the approximations made on the coefficients. The only way to reduce this approximation is to increase the value of $n_{0}$. Since this paper does not aim at the characterization of the digital filter, we here only state that this phenomenon has been simulated on Matlab^®^, following the sketch from Figure 5 with the same parameter values. Increasing $n_{0}$ up to $n_{0}=40$ leads to frequency responses much closer in amplitude than the theoretical ones, which has also been experimentally validated.

### Appendix B.3. Correlated Noise

Another phenomenon occurring within the digital filter is its unexpected variable output amplitude: it can be clearly seen on the GUI oscilloscope that the output amplitude is varying over a long period of time in comparison with the period of the signal. It was found that these variations are due to the approximation done by the right bit-shifting of block 36: the number of digits on which is encoded the feedback signal in the IIR has an influence on the time response. In order to reduce the influence of this function on the response, the signal is multiplied by another factor $2^{n_{1}}$ at the output of the FIR, and is divided by the same constant at the output of the IIR (blocks 20 and 32). This way, the feedback (between blocks 37, 24, 26 and 28) is coded on 45 bits and not on 20 since $n_{1}$ can go up to 25. This additional floating point method enables to reduce the correlated noise in the IIR. This way, for $n_{1}=10$ and $n_{0}=40$, the relative error of the output amplitude of the digital filter is less than 0.5% compared to that of the analytical model.
